# Positive nutrition: shifting the focus from nutrients to diet for a healthy lifestyle

**DOI:** 10.1007/s40519-023-01580-1

**Published:** 2023-06-21

**Authors:** Daniela Martini, Hugo da Costa Ribeiro, Paul Gately, Richard Mattes, Roberta Re, Dennis Bier

**Affiliations:** 1 Department of Food, Environmental and Nutritional Sciences (DeFENS), University of Milan, 20122 Milan, Italy; 2 Department of Pediatrics, Federal University of Bahia School of Medicine, Salvador, Bahia, 40110-060 Brazil; 3 Carnegie Research Institute, Leeds Metropolitan University, Leeds, LS6 3QS UK; 4 Department of Nutrition Science, Purdue University, West Lafayette, IN 47907 USA; 5 Cambridge Food Science, Cambridge, CB23 5AB UK; 6 Children’s Nutrition Research Center, Baylor College of Medicine, Houston, TX 77030, USA

**Keywords:** Nutrients, Dietary pattern, Obesity, Mediterranean diet

## Abstract

**Purpose and methods:**

This summary is based on a scientific symposium organized by the Mediterranean Diet Roundtable and the American Italian Food Coalition titled, *‘Positive Nutrition: shifting focus from nutrients to diet for a healthy lifestyle.*’ It was held at the Embassy of Italy in Washington DC in September of 2022. The panel of experts discussed how science can inform policy, what insights may be gleaned from different countries’ approaches to healthy eating and what principles of the Mediterranean diet will inform strategies for a healthy future. Recognizing that isolated actions have limited impact on the complex relationship between diet and obesity, the panel discussed the importance of a system approach. In particular, the panel emphasized that focusing on single ingredients, isolated food categories and narrow approaches to policy have had limited success across the globe.

**Results and conclusion:**

The panel agreed that there is a need for change of perspective that embraces complexity and emphasizes more positive nutrition messaging and policies.

*Level of evidence*: V, Opinions of respected authorities, based on descriptive studies, narrative reviews, clinical experience, or reports of expert committees.

## Introduction

With the discovery of essential nutrients in the first half of the twentieth century, nutritionists began studies aimed at elucidating the roles of nutrient balances and intake of selected foods in disease prevention. Over time, it was realized that these efforts were hampered by a number of factors: (1) people eat complex foods, not individual nutrients; (2) observational studies are unable to determine causality; (3) challenges to the design, implementation and funding of long-term intervention trials; and (4) innumerable unknown and unaccounted-for environmental covariates that confound interpretations of any outcomes. Thus, the feasibility and interpretation of long-term feeding trials is problematic. In response, an alternate approach to inquiry of the relationships between diet and chronic disease prevention has emerged that assesses the nutritional basis of varied traditional diets consumed in different regions of the planet. This work has emphasized the importance of food matrix interactions, lifestyle and societal variables, and environmental conditions contributing to long-term health status. Moreover, this perspective provided a rationale for moving away from considering only the conventional reductionist approach to understanding these relationships, an approach often focused negatively on “what not to eat,” rather than on “what to eat”. Doing so also lead to new analytical methods for assessing the complexities of whole diets and individual lifestyles that support positive approaches to long-term health maintenance. This shift in emphasis is distinctly more relevant for, and likely palatable to, the general public. Going forward, research must clarify the interdependencies of diet, lifestyle and environment and their contributions to causal relationships among the foods we eat and disease prevention while recognizing that eating well should not, and does not, mean giving up the pleasures of eating. Any food, in appropriate portion and frequency of consumption, can be part of a varied and balanced diet. The goal is to select enough of the right sorts of food to meet nutrition requirements while maintaining a healthy weight and retaining the pleasures of eating. This is a critical shift in narrative from exclusion to inclusion, helping people move away from punitive, guilt-tainted consumption rules to sustainable, positive, long-term lifestyle habits.

The core concept of Positive Nutrition is to encourage consumption of healthy nutritious foods rather than demonize consumption of less nutrient-dense alternatives. Thus, the point of this narrative is to emphasize the importance of avoiding a focus on individual nutrients in isolation while refocusing on proven food consumption patterns, specifically what we can learn from regional diets (e.g., Mediterranean, Nordic, Blue Zone) [1, 2], how this information can be applied to Policy, and how lifestyle and eating behaviours should be taken into consideration in policies setting obesity strategies. Perspectives were presented at a scientific symposium organized by the Mediterranean Diet Roundtable and the American Italian Food Coalition at the Embassy of Italy in Washington DC in September of 2022. The theme of the meeting was the impact of communication of science in dietary guidelines and policies with the title ‘Positive Nutrition: shifting focus from nutrients to diet for a healthy lifestyle’ taken place.

Building on this perspective, speakers at this symposium discussed how public policy can be built upon, and help support, recommendations based on whole diets, physical activity, and associated healthy lifestyles. With the convening of the White House Conference on Hunger, Nutrition, and Health in September, the 2025 Dietary Guidelines now under review, and the Farm Bill set for reauthorization next year, policymakers have a full agenda. This symposium aimed to provide a rationale for adoption of positive nutritional strategies for a healthy future. Good nutrition is about health maintenance and chronic disease prevention through appropriate consumption of all known essential nutrients and the other, as yet uncharacterized, non-nutrient food components that appear to be protective against disease development.

### Nutrition and health or nutrients and health?

Categorizing foods by individual nutrient composition neglects the contribution of the whole food to the total diet and sacrifices the synergistic roles of nutrients with other food constituents. Moreover, policy messaging is often polarized between healthy and unhealthy foods (good foods vs bad foods). This dichotomy is false and misleads the consumer, since food choices are not simply binary. Oversimplifying a complex story no longer is an adequate or suitable approach to dietary recommendations.

The importance of the relationships among individual nutrients within foods, the dietary patterns in which foods are consumed and the integrated consequences of these variables and long-term human health represents one of the most current, important, and hotly debated issues in the field of human nutrition. The ultimate goal of nutrition is to provide the body with the nutrients essential for meeting its requirements. It is equally important that foods, irrespective of their nutrients, must be eaten in amounts that promote maintenance of healthy energy balance. Nutrition science has long-focused on the health risks associated with the consumption of excessive amounts of dietary energy, principally as the macronutrients of fats and carbohydrates. However, this focus has been questioned by recent literature supporting an approach based on the principle of “positive nutrition” that promotes the consumption of suitable quantities of foods and food groups in healthful balances rather than advocating for limiting the intake of specific macronutrients or foods, let alone food groups [3].

Applying a hierarchy of choosing diet over nutrient also changes the focal point of several public health tools. For example, nutrition claims that highlight specific nutrients or single dietary components (e.g., low-fat, “light”) have a long history of use [4] but have clearly not been able to reduce the adverse health burden attributable to overnutrition. Such claims contribute to the misperception of the “healthiness” of products which often have only marginally better nutrition profiles than those without claims [5, 6]. Likewise, nutrient profiling systems are tools often used to target marketing to vulnerable segments of the population. This approach has been criticized by European Food Safety Authority (EFSA) and other agencies for a decade or more [7]. The critiques have been directed at the use of arbitrary, not evidence-based, intake “cut-offs” that do not consider the variable needs for different people. Additionally, the first and foremost limit may be its apparent goal of “balancing” the nutritional composition of each individual food rather than considering individual foods as parts of a dietary pattern composed of multiple food items with different characteristics and composition. In this scenario, it is not necessary that each individual food, per se, be balanced, a principle that is not supported by the scientific evidence. No individual food contains all the essential nutrients and no matter how health-promoting many foods are when consumed in whole diets, individual foods can never themselves satisfy all the criteria of being “healthy”. Olive oil might serve as a readily apparent example. Rather, there is a need to focus on the many and varied combinations of different foods that lead to a healthy dietary pattern. Such is the case for the Mediterranean dietary pattern which comprised different foods in each of the nations in the Mediterranean region. Each includes the consumption of individual foods that would themselves be “penalized” by some nutrient profiling systems despite their demonstrably positive nutritional role in whole, yet variable, dietary patterns. In a similar way, profiling systems often base their food values on consumption of fixed amounts of foods (e.g., 100 g) regardless of customary portion sizes, a construct that leads to penalizing foods normally consumed in smaller portions, or only occasionally, such as chocolate and olive oil [8]. Overall, then, the risk of such artificial, subjective constructs, often founded on “allegiance biases” rather than objective evidence, is to profoundly oversimplify and corrupt nutrition public health messaging. While the commendable intention might be to deliver clear and simple messages to the consumer, this approach leads to false conflicts between “healthy” and “harmful” foods that may contribute to increased confusion and lack of efficacy.

On the other hand, dietary guidelines for healthy eating should not limit consumption of any food products, but specify recommended amounts for an enjoyable diet that is both varied and balanced. It is a food culture based on awareness rather than restriction, with core principles that are geographically transferable.

### Obesity, a too simple approach to a complex problem

Preventing and managing overweight and obesity are complex problems, with no simple answers. When designing dietary recommendations to influence levels of obesity, consideration of the wider influences, complexity, unintended consequences (such as weight stigma) and to recognize that multiple coordinated action is necessary. Many of the Public Health Policies/actions from Healthy People 2030 and the Centers for Disease Control and Prevention (CDC) recommendations [9] continue to promote traditional isolated and simplified/singular actions on food and physical activity, with many also promoting the continuation of a restriction/reduction approach while the incidence and prevalence of obesity have continued to rise. However, past experience indicates isolated ingredients or isolated actions hold limited impact on obesity. The current Healthy people strategy for 2030 [10] and the recent White House Strategy on Hunger, Nutrition and Health [11] understandably both focus efforts on nutrition and physical activity, the key behaviours in energy balance. There has also been progress recognizing the importance of food insecurity as a relevant and current driver of health issues, further widening the complexity of the determinants of health. Recently whole systems approaches have emerged as a tool to tackle complex or ‘wicked’ problems like obesity and food intake. For example, the Food and Agriculture Organization (FAO) recognizes the importance of systems thinking in its vision for sustainable food systems [12]. Such an approach seeks to move beyond binary thinking (good food vs bad food; good actors vs bad actors); and linear thinking (cutting sugar will reduce obesity), and thinking our world is stable and predictable (COVID revealed we live in a very dynamic world).

### The Mediterranean Diet Model to foster behaviour change

The Mediterranean model, which varies markedly between nations in the region, is fundamentally based on a plant-based dietary pattern (5 portions a day of vegetables and fruits, 4 portions a day of whole grains, 3 spoons per day of extra-virgin olive oil for a 2000 kcal diet) with a small contribution of animal source foods (1 portion a day of meat or fish or eggs, 2 portions a day of milk or dairy products) combined with other cultural characteristics (moderation/frugality, conviviality, has activities, local and regional foods, adequate rest and physical activity) [13]. These elements represent the reason why UNESCO has considered the Mediterranean diet an intangible cultural heritage since 2011.

Evidence supports a Mediterranean style diet for the prevention of non-communicable diseases (cardiovascular and metabolic diseases, cancer, depression) [14, 15]. This has been attributed to its moderating effect on insulin sensitivity and glycaemia, reduction of inflammation, protection from oxidative stress, and epigenetic effects that favourably influence the expression of genes contributing to development of the above-mentioned diseases [16]. A positive nutrition approach should enhance consumption of balanced diets, while a concomitant lifestyle educational intervention will also improve sustainability of a reinforcing health lifestyle [17].

### How can science inform policy?

Eating habits and lifestyles have changed markedly in the developed world. Increased food intake due to both increased potions sizes [18–20] and eating frequency [21–23] as well as reduced physical activity [24–26] are among the most notable trends. This has coincided with an increase in non-communicable diseases linked to lifestyle factors such as obesity, diabetes, and cardiovascular diseases.

Policies often focus on reducing consumption of certain foods (i.e. high fat, sugar and salt HFSS). In many cases, this has had no effect or been associated with behavioural changes that were actually counter-productive. The population response to messaging about avoidance of high fat foods is a clear example [27] as such guidance led to increased consumption of low-fat foods that more than offset the energy savings achieved by reducing the fat content of foods. Indeed, the incidence of overweight and obesity continued to grow when the food supply was enriched with low-fat products. Another example is the targeting of recommendations to reduce added sugars. This has resulted in some reduction of their intake, but there has been an offsetting increase in saturated fat intake in the population with the result of no change in energy intake [28]. Another complication of promoting exclusion of selected foods is that while they may add a certain amount of energy, they may also be a substantive contributor of desired nutrients [28]. For example, food sources of added sugars can also be important contributors of the daily intake of whole grain in the diet [29–31]. Similarly, exclusion of ultra-processed foods may compromise diet quality since many of these produces are enriched and fortified [32, 33]. A third complication with exclusion recommendations is that they may lead to poor overall diet acceptance and lack of adherence [34]. No diet will work if it is not followed.

A prerequisite for effective dietary change is that adherence to the diet be feasible on a sustained basis. Restriction diets often violate this principle. In contrast, diets best positioned for success, emphasize balance, moderation and variety. Their premise is that all foods may be incorporated into a healthful diet when consumed in appropriate portions and at appropriate frequency.

Given these concerns, where can the evidence to support decisions by policy makers, clinicians and the general public, be found? Unfortunately, given the limitations of current scientific approaches to this question, direct answers about specific foods are unlikely to be forthcoming in the immediate future. Observational studies are problematic because they cannot accurately and precisely measure food intake, are generally deficient in estimating food composition, cannot account for the numerous co-variate confounders and, most importantly, cannot determine cause and effect. Interventional studies are likewise problematic because it is almost impossible to design diets where one can manipulate only a single food or nutrient. Moreover, unambiguously testing the intervention is almost always inadequate because of failure of long-term dietary compliance. Even so, rarely is sufficient funding available to conduct such interventional trials in a sufficient number of subjects over the prolonged periods of time required to convincingly answer any questions of chronic risk.

For these reasons, in recent years, scientists have turned to a more generalizable, encompassing approach that aims to capture the full extent of population dietary habits developed over centuries to health outcomes seen over the corresponding periods of time. In a sense, then, the approach aims to integrate all the historically available information contained in traditional, regional dietary patterns with long-observed population health outcomes. Although regional dietary patterns are available in different areas of the globe such as the Nordic countries [35–38] and Asian regions [39–41], by far the most extensive data have been collected and studied in the countries that form the geographical boundaries of the Mediterranean Sea, resulting in the now widely accepted nutritional principles labelled the “Mediterranean Diet” [42, 43].

The advantages of this dietary pattern approach are many. First, the health promotion outcomes are well established and widely reported [44]. Secondly, the foods that constitute the diets are widely available throughout most geographical regions as they have been, generally for centuries. Third, all foods historically found in the established dietary pattern are familiar and acceptable, so the “negative” stigma associated with many nutrition recommendations are not incorporated into public health policy or education. Thus, the consumer does not require instruction in artificial constructs such as nutrient profiling systems, product colour-coding, package stoplights, nutrients of concern labelling, and related schemes.

Over the course of approximately the last half-century, nutritional recommendations and policies have become increasingly more complex in an attempt to better answer the question of *“what should I eat to better maintain my health?*” It is no longer clear that this complexity of public information transfer has, in fact, resulted in the intended health outcomes for the reasons discussed above. Meanwhile, the approach has unintentionally confounded simple nutritional messages intended for the public.

## Data Availability

Not applicable.
